# A comparative study evaluating three line immunoassays available for serodiagnosis of equine Lyme borreliosis: Detection of *Borrelia burgdorferi* sensu lato-specific antibodies in serum samples of vaccinated and non-vaccinated horses

**DOI:** 10.1371/journal.pone.0316170

**Published:** 2024-12-23

**Authors:** Cornelia V. Broeckl, Stephanie Hiereth, Reinhard K. Straubinger

**Affiliations:** Faculty of Veterinary Medicine, Department of Veterinary Sciences, Institute for Infectious Diseases and Zoonoses, Chair of Bacteriology and Mycology, Ludwig-Maximilians-Universität Munich, Oberschleißheim, Bavaria, Germany; Berhampur University, INDIA

## Abstract

Diagnosis of equine Lyme borreliosis (LB), an infection caused by members of the *Borrelia burgdorferi* sensu lato complex (*Bb*sl), is challenging due to the nonspecific clinical signs of the disease and due to the variety of non-standardized serological tests. Specific vaccine-induced antibodies against LB, providing an effective protection against the infection, complicate the issue further. The standard for the detection of specific antibodies against *Bb*sl is a two-tier test system based on an enzyme-linked immunosorbent assay (ELISA) or indirect fluorescent antibody test (IFA) for antibody screening combined with a qualitative, highly specific immunoassay (e. g. line immunoassay (LIA)) for confirmation. In this study, three LIAs available for detection of antibodies in equine serum samples were evaluated and compared. A total of 393 serum samples of 131 horses with known serostatus were used. It included groups of non-vaccinated horses, immunized horses (vaccinations against LB on days 0 and 14), and horses that had received an initial immunization plus an additional booster on day 180. Sera were collected on days 0, 135 and 210 of the study. Results were compared considering the tests’ sensitivity, specificity, diagnostic outcome, and the operability of each test. Agreements of the diagnostic results among the LIAs were calculated for overall test results and single antigen-antibody-complex signal results. They are presented as inter-rater agreement and statistic reliability, represented by the Fleiss’ kappa coefficient. Agreement scores ranged from poor to moderate depending on group and time-point of blood sample collection. Depending on LIA used, deficiencies were observed in the form of non-sufficient sensitivity of antigen signals on the LIA strips (especially for outer surface protein A (OspA) or variable major protein like sequence expressed (VlsE)) or as an inappropriate test interpretation of the OspA signal. Operability of the three LIAs was equally user-friendly with minor variations. In two LIAs, test-evaluation was simplified by a supplied scanner and evaluation software. To improve functionality of available LIAs for equine serum samples it is advisable to adjust sensitivity and specificity of single test antigen signals and establish appropriate evaluation protocols.

## 1. Introduction

Due to more favorable climate conditions the likely geographic range, the distribution and abundance of ticks of the *Ixodes* family, the vector of borreliae from the *Borrelia burgdorferi* sensu lato complex (*Bb*sl), is increasing [1]. Especially rural areas with large natural and seminatural greenspaces, a low field vegetation height and a high vertebrate host spectrum bear risk for an increased density of tick populations, particularly during summer months [2, 3]. Regarding tick-borne diseases (TBD) such as Lyme borreliosis (LB), or Lyme disease (LD), an increasing emergence and a shift in seasonality can be observed [4, 5]. Although there is sparse data available on the seroprevalence and clinical presentation of LB in horses in Europe, horses are potentially at extended risk of tick encounter, transmission and infection with TBD [6, 7]. Focusing on equine LB, the pathogenesis is not well elucidated, however, infection and clinical disease are two different entities. Seropositivity itself is not associated with clinical disease and, accordingly, a single positive serologic test is not enough for a confirmation of LB in horses. Yet, a serum sample with *Bb*sl-specific antibodies might be evidence of a current or past exposure to borrelia antigens (AG). Documented clinical signs in horses are manifold, but generally unspecific, and include fever, stiffness, intermittent lameness, decreased performance, and behavioral changes due to encephalitis, uveitis, infectious arthritis, neurological disease or cutaneous pseudolymphoma [8–10]. The diagnosis of LB should only be confirmed if differential diagnoses have been eliminated and the criteria of tick exposure, the manifestation of clinical signs and the detection of the pathogen in the patient, or a specific antibody response, apply [11].

To prevent an infection with *Bb*sl, vaccines are available for horses, yet, to ensure a protective effect it is necessary and recommended to perform two initial immunizations within 2–3 weeks, a third immunization 6 month later, a fourth immunization one year after the first immunization, and then repeat the vaccination on an annual basis prior to the peak of tick activity [12, 13]. The lysate vaccines available contain inactivated borreliae antigens, including the outer surface protein A (OspA). Vaccination-induced immunoglobulins are taken up by the tick during its blood meal. In the tick’s midgut, antibodies bind to the expressed OspA antigen on the borreliae. Antigen-antibody binding results in the immobilization of the spirochetes, inhibiting the spirochetes’ migration to the tick’s salivary glands and their injection into the host’s skin [14, 15].

To confirm *Bb*sl infection and strengthen clinical diagnosis, it is essential to have operable and reliable serodiagnostic tests for the detection of antibodies (AB) specific for an ongoing infection with *Bb*sl. In this context, it is of great importance that tests allow the differentiation of vaccination-induced AB from those induced by past, natural exposure and active infection. Only horses with clinical signs consistent with LB and infection-specific antibodies against *Bb*sl need antibiotic treatment; while a false-negative serodiagnostic result may entail the omission of appropriate on-time treatment, a false-positive result may evoke unnecessary antibiotic treatments with detrimental effects on the horses’ health (e.g. effects of drug intolerance, colitis, diarrhea, increased risk for additional infections) and the risk of antibiotic-resistance development in bystander bacteria [16]. However, diagnosis of LB in horses is challenging; recommended serological standard methods often fail at this challenge impeding clinical decision-making and potentially leading to misdiagnosis and mistreatment of the herbivorous patient.

Today’s method of choice are non-standardized indirect tests detecting *Bb*sl-specific AB in serum; it is a two-tier test system based on an enzyme-linked immunosorbent assay (ELISA) or indirect fluorescent antibody test (IFA) for screening for reactive antibodies combined with a qualitative and highly specific immunoassay (e. g. line immunoassay (LIA)) for confirmation. Test strips of immunoblots are coated with antigens specific for member species of the *Bb*sl complex. Suitable and commonly used antigens for immunoblotting are decorine binding protein A (DbpA, p18), outer surface protein C (OspC), outer surface protein A (OspA, p30), *Borrelia* membrane protein A (BmpA, p39), protein 100 (p100, p83), protein 58 (p58), peptid C6 and variable major protein-like sequence expressed (VlsE). If specific AB are present in the sample, they bind to its specific AG on the test strip resulting in AG-AB immunocomplexes which are visualized by color reaction signals on the strip [17, 18]. Semi-quantitative LIAs are highly specific potentially allowing a differentiation between naturally infected and vaccinated animals. Due to the vaccination with LB lysate vaccines, animals develop a humoral OspA-specific IgG-response with antibody levels depending on the vaccination schedule and time of testing [12, 19]. Thus, reactions to OspA antigen is a consistent marker for immunization against LB [20]. Yet, although a reaction to OspA AG in LIA is considered as indicative for vaccination–also in equine serum samples–and most test kit manufacturers supply instruction manuals accordingly, informative LIA evaluation protocols for serum samples of vaccinated horses covering this state of knowledge have not officially been published to this date. Additionally, it needs to be considered that the use of lysate vaccines against LB in horses might result in the development of antibodies against diverse other borrelial outer surface proteins that are expressed by borreliae in *vitro* (i. e., OspC, DbpA, BmpA, p58, or p100). That is, the appearance of respective immunocomplex reaction signals on LIA strips in combination with a positive reaction to OspA AG commonly occur in vaccinated animals and need to be considered as vaccination-induced and not be counted as infection-specific signals. In contrast, VlsE and C6 AG signals are considered as highly specific markers for a *Bb*sl-infection since those are only expressed by viable borreliae in *vivo* [21–23].

In this project we compared and evaluated three LIAs available for detection of AB against *Bb*sl in equine serum samples with focus on their ability to differentiate antibody responses initiated by immunization and natural infection. A total of 393 serum samples of 131 clinically healthy horses with known serostatus were used. The sample-pool originated from a vaccination study and included groups of non-vaccinated horses, basic-immunized horses (vaccinations against LB on days 0 and 14), and horses that had received immunizations against LB plus an additional booster on day 180 [12]. Sera were collected on day 0 (prior to first vaccination), as well as on days 135 and 210 in the vaccination schedule since differences in diagnostic results (specific antibody levels) are expected between experimental groups at these time-points. Tests were compared regarding their sensitivity, specificity and diagnostic outcome, and were evaluated for handling, operability and evaluation procedure to identify individual benefits and deficiencies of a test. Moreover, with the aim to refine serodiagnostic accuracy, a recommended overall evaluation protocol (ROEP) for serodiagnosis of LB was established as an up-to-date reference protocol for the evaluation of equine serum samples when LIAs are used.

## 2. Material and methods

All procedures for this study were carried out in accordance with the guidelines approved by the Animal Welfare Committee of the Government of Upper Bavaria, Munich, Germany (Az. 55.2.1.54–2532.0-91-16) and the protocols adhered to the German ‘‘Tierschutzgesetz” (§ 8a Abs. 1 Nr. 3b TierSchG).

### 2.1. Serum samples

A total of 393 equine serum samples were analyzed for the presence of specific antibodies against *Bb*sl using LIAs produced by three different manufacturers (S2 Table). The sera were originated from a previous vaccination study with horses using the vaccine EquiLyme® (Boehringer Ingelheim Vetmedica GmbH, Ingelheim am Rhein, Germany [12]) and were stored at -80°C. All equine serum samples described in this study have been tested in advance for quantitative and qualitative antibody levels with in-house kinetic ELISA (KELA) and LIA and therefore had known serostatus [12].

The collection of sera consisted of samples collected from a total of 131 clinically healthy horses at three time-points in the vaccination schedule and included one group of non-vaccinated and two groups of vaccinated horses that underwent variant vaccination schedules (S1 Table). Horses in group Non-Vac (*n =* 42) received no vaccination and functioned as the negative control group. Horses assigned to group Vac-Basic (*n =* 45) were vaccinated following a basic vaccination schedule which included two vaccinations: on day 0 (first immunization) and day 14. Horses belonging to group Vac-Plus (*n =* 44) were vaccinated equally to study group Vac-Basic, yet, had received an extra booster on day 180. For this study we used sera collected from each participating horse on sampling time-point day 0 (d0), day 135 (d135) and day 210 (d210) in the vaccination period. We chose this specific selection of study groups and blood sampling time-points to demonstrate that, depending on the time-point of the latest performed vaccination, there may be significant differences in equine immune responses. This comprises a major pitfall in the serodiagnosis of equine LB, particularly with regard to differentiating vaccinated and naturally infected horses.

Additionally, a serum sample of a *Bb*sl-infected and LB-vaccinated horse with known, high seropositivity was included as positive control (RKS-B/9940-E) in every single test run.

For the serologic testing, the sera were thawed twice and either stored at +**4**°C for a maximum of five hours between two tests or refrozen right after usage.

### 2.2. Antibody detection in the serum samples

Serum samples were tested each with three different line immunoassays further named LIA A, LIA B and LIA C (S2 Table).

The LIA strips are coated with antigens specific for *Borrelia* species of the *Bb*sl complex. The detection of *Bb*sl-specific antibodies in equine serum with the supplied LIAs was carried out according to manufacturer’s instructions as described in the following section (and S1 Fig). Only test kits with one and the same LOT number per manufacturer were applied to test all samples in order to exclude the possibility that the use of different LOTs may have influence on test results. Samples were not tested according to the study groups but were processed randomly.

Depending on the technical instruction manual of the LIA applied, the evaluation of the LIA strips was conducted either visually by the examiner (i. e. LIA A) or automatically by a scanning and evaluation software (i. e. LIA B and LIA C).

Evaluation results written in small letters (i. e. “negative” (neg), “equivocal positive” (equivoc), “positive” (pos)) refer to a result of a single AG line (single color reaction signal result) on a test strip. Evaluation results written in capital letters (i. e. “NEGATIVE” (NEG), “EQUIVOCAL” (EQUIVOC), “POSITIVE” (POS), “VACCINATED” (VAC), “VACCINATED AND POSITIVE” (VAC+POS)) refer to the cumulative diagnostic outcome (overall test result) of an entire test strip or serum sample. Yet, the expressions “false-positive” or “false-negative”, as used several times in this publication, refer to the misdiagnosed or misclassified, cumulative diagnostic outcome of a test.

#### 2.2.1. LIA A

LIA A is a line immunoassay developed for semi-quantitative detection of IgG antibody response against antigens that are specific for *Borrelia* species of the *Bb*sl complex. It was designed for testing of equine and canine sera. To test equine serum samples, though, it is required to acquire both, the canine test kit plus the supplemental equine set, to have all necessary test components available. The serological testing was conducted according to manufacturer’s instructions with supplied nitrocellulose strips coated with *Bb*sl-specific antigens and provided reagents. Antigens present on the strips are listed in Table 1. For every test kit one equine IgG cut off control (COC), a serum sample supplied as concentrate by the manufacturer, needs to be included in every single LIA run. COC were generally handled like every other serum sample but were always placed in the first reaction well of the incubation tray and, later, on a separate evaluation sheet. The COC was developed to function as coloration reference for the signal intensity of AG-AB immunocomplex reactions. During the evaluation process it is compared to signals on the test strips evoked by serum samples tested.

**Table 1:** *Borrelia burgdorferi* senso stricto-specific antigens applied on nitrocellulose strips of three LIAs used.

Antigen	LIA A	LIA B	LIA C
VlsE	+	np	+
VlsE-Bb*	np	+	np
VlsE-Ba**	np	+	np
C_6_-Peptid	np	np	+
OspA	+	+	+
DbpA (p18)	+	+	+
ErpA-p18***	np	+	np
OspC	+	+	+
BmpA (p39)	+	+	+
p58	+	+	+
p100 (p83)	+	+	+
p41	np	np	+
L-Bb*	np	+	np

LIA, line immunoassay; *Ba*, *Borrelia afzelii*; *Bb*, *Borrelia burgdorferi*; VlsE, variable major protein-like sequence expressed; C6, peptide referring to 6^th^ invariant region of VlsE; Osp, outer surface protein; DbpA, decorine binding protein A; ErpA, A-type iron sulfur protein essential for respiratory metabolism in *Escherichia coli;* BmpA, *Borrelia* membrane protein A; p, protein; L, lipid; antigens present (+) and not present (np) on the assay; *antigen on strip originated from *Bb*; **antigen on strip originated from *Ba*; ***according to the manufacturer the recombinant protein ErpA-p18 is of different origin than DbpA (p18) on their LIA strip and therefore unequal.

As instructed by the manufacturer`s manual, all chemical components utilized for antibody detection with LIA A were brought to room temperature before use.

Then, the washing and incubation buffer (WB) was prepared in a ratio of 1:10 by mixing concentrated buffer with a respective volume of distilled water (*Aqua destillata* (*Aq*. *dest*.)). For each serum sample to be tested one nitrocellulose test strip was placed in a reaction well of an 8-well incubation tray. Then, 1.5 ml of WB were pipetted into each well used and the incubation tray was swayed gently to fully moisten the test strips. This step was followed by the addition of test samples: in the first reaction well 100 μl of COC was added, in the second and further wells 15 μl of an equine serum sample were added to an assigned well and test strip. The incubation was performed at room temperature for 30 min on a rocking shaker. Subsequently, the fluid was gently poured off while test strips remain in the well, replaced by another 1.5 ml of WB and incubated for 5 min on the rocking shaker; this 5-minute washing-and-draining-step was repeated two more times. Meanwhile, the anti-horse IgG-conjugate-dilution was prepared in a ratio of 1:100 by mixing equine conjugate concentrate with a respective volume of WB. Then, 1.5 ml of conjugate-dilution was added to each drained well with a test strip, followed by an incubation period of 30 min on the rocking shaker and, after the removal of all fluids, three further 5-minute washing-steps with 1.5 ml WB each. After finishing the washing-and-draining process, 1.5 ml of *Aq*. *dest*. was added to each well for 1 min, then poured off, replaced by 1.5 ml of substrate per well and incubated for exactly 12 min on the incubation shaker. The test was finalized by one last draining-step and three rinsing-steps with 1.5 ml *Aq*. *dest*. each without incubation. At the end, all fluids were removed, the strips carefully transferred from the wells to a clean absorbent tissue and left to dry for at least 20 min away from sunlight. For evaluation, the LIA strips were first arranged and attached one below the other on an evaluation sheet–with an extra sheet for the COC.

Evaluation of the strips was conducted visually by the examiner. Color reactions on single antigen lines were categorized with values 1 (negative (neg); no signal), 2 (negative (neg); weak signal fainter than COC), 3 (equivocal positive (equivoc); signal equal to COC), and 4 (positive (pos); signal stronger than COC). The overall rating of an entire test strip or serum sample in LIA A and its assignment to an overall test result allocation category is explained in Table 2. Hereby, the manufacturer of LIA A distinguishes between the allocation categories “NEGATIVE” (NEG)–no evidence of relevant contact with pathogen, “EQUIVOCAL”–evidence of contact with pathogen, “POSITIVE” (POS) (infection)–evidence of infection with pathogen. The OspA AG line was not considered as a specific AG signal in equine immunoreactions. Thus, there is no differentiation and specification as vaccination or both as vaccination and infection.

**Table 2:** LIA A–overall rating of a test strip or equine serum sample and its assignment to an overall test result allocation category according to manufacturers’ instructions.

Allocation categories (overall test result)	Antigen line evaluation—Equine sera
**NEG**	0 AG lines or AG lines < COC or 0–2 AG lines ≥ COC (except VlsE)
**EQUIVOC**	VlsE AG line and 0–2 AG lines ≥ COC or 3 AG lines (except VlsE) ≥ COC
**POS**	VlsE AG line and ≥ 3 AG lines ≥ COC or VlsE AG line and DbpA AG line and ≥ 1 AG line ≥ COC or ≥ 4 AG lines ≥ COC (except VlsE)
**VAC**	-
**VAC+POS**	-

Overall rating and allocation are based on the AG line results obtained with LIA A and its evaluation according to LIA A’s evaluation protocol for horses supplied by the manufacturer. The OspA AG line was not considered as a specific AG line for an equine immunoreaction. Hence, in this protocol there is no differentiation in terms of a vaccination (VAC) or vaccination and infection (VAC+POS) regarding an overall test result.

AG line, antigen line; NEG, negative overall test result; EQUIVOC, equivocal overall test result; POS, positive overall test result (infection); COC, cut off control; OspA, outer surface protein A; VlsE, variable major protein-like sequence expressed; DbpA, decorine binding protein A; COC, cut off control.

#### 2.2.2. LIA B

Other than LIA A, LIA B was designed for testing equine serum samples only. Antigens present on the strips are listed in Table 1. Antibody detection was performed according to the manufacturer’s instructions. Evaluation of the strips was conducted with the software and scanner system supplied by the manufacturer. In case of this LIA, the COC was already included on the test strip and no additional procedure was needed in this context.

LIA B was prepared and conducted alike LIA A with the following differences: in LIA B 30 μl of serum samples were used, incubation trays held 30 wells, and strips were required to be incubated in advance for 15 min in 1.5 ml of WB-dilution before adding serum samples. The subsequent steps of incubation of serum samples and conjugate (30 min each) as well as the two washing steps in between (3 x 5 min each) were equal to LIA A. However, in LIA B the anti-horse enzyme-conjugate-dilution was prepared in a ratio of 1:10. After finalizing the second washing process with WB, no further washing step with *Aq*. *dest*. was conducted, but the substrate was applied immediately, and incubation was performed for only 10 minutes. To stop the color reaction fluids were poured off and strips were washed-off with 1.5 ml *Aq*. *dest*. three times for 1 min each. After finishing the test, transferring strips to a prepared sticky evaluation sheet and air-drying them for at least 20 min away from sunlight, evaluation was performed using a flatbed scanner and evaluation software (version 3.4.36) supplied by the manufacturer.

Color reactions on single AG lines were evaluated and categorized automatically by the software with values from 0 to 11 (negative (neg); no signal), 12 to 18 (equivocal positive (equivoc); very weak signal or equal to COC), and ≥ 19 to 255 (positive (pos); medium to strong signal greater than COC). The overall rating of an entire test strip or serum sample in LIA B and its assignment to an overall test result allocation category is explained in Table 3. Hereby, the manufacturer of LIA B distinguishes between allocation categories “NEGATIVE (NEG)–no evidence of relevant contact with pathogen”, “EQUIVOCAL (EQUIVOC)–serological evidence of contact with pathogen”, “POSITIVE (POS)–serological evidence of infection” and “VACCINATED (VAC)–serological evidence of immunization”. Thus, the OspA AG signal is considered as specific AG signal in equine immunoreactions. In the software there is an additional allocation category for the case of both, a vaccination and an infection, classified as “VACCINATED OR POSITIVE (VAC+POS)–Positive—serological evidence of infection or immunization”, but with no further explanation for the type and number of AG lines.

**Table 3:** LIA B–overall rating of a test strip or equine serum sample and its assignment to an overall test result allocation category according to manufacturers’ instructions.

Allocation categories (overall test result)	Antigen line evaluation—Equine sera
**NEG**	0 AG lines or AG lines < COC or 1 AG line > COC or p100 AG line and 1 AG line > COC
**EQUIVOC**	2 AG lines > COC (except p100) or 2 AG lines > COC and ≥ 1 AG line equivoc or 1 AG line > COC and ≥ 2 AG lines equivoc or ≥ 3 AG lines equivoc
**POS**	≥ 3 AG lines > COC (except OspA)
**VAC**	OspA AG line and other possibly positive AG lines
**VAC+POS**	- *

Overall rating and allocation are based on the AG line results obtained with LIA B and its evaluation according to LIA B’s evaluation protocol for horses supplied by the manufacturer. VlsE-*Ba* and VlsE-*Bb* were always considered as one AG line albeit both AG lines might appear as “positive “or “equivocal positive”. The OspA AG line was considered as specific AG line in equine immunoreaction. Hence, there was an overall test result allocation category for vaccinated animals (VAC, vaccinated). *Only in the manufacturer’s evaluation software there is an additional allocation category for the case of both–a vaccination and an infection (VAC+POS)–classified as “Positive—serological evidence of infection or immunization”, but with no further specification regarding the type and number of AG lines.

AG line, antigen line; NEG, negative overall test result; EQUIVOC, equivocal overall test result; POS, positive overall test result (infection); equivoc, equivocal positive AG signal result on single AG line COC, cut off control; OspA, outer surface protein A; p100, protein 100.

#### 2.2.3. LIA C

Alike LIA A, LIA C was developed to test equine and canine serum and plasma. Yet, a kit included components for testing sera of both species. All antigens present on the strips of LIA C are listed in Table 1. Antibody detection was performed according to manufacturer’s instructions. Evaluation of the strips was conducted with the software and scanner system supplied by the manufacturer. In this LIA the COC was already included on the test strip.

LIA C was prepared and conducted like LIA A and LIA B with the following differences: the WB-dilution was prepared in a ratio of 1:5, incubation trays held 10 wells each and pre-incubation of strips in WB was performed for 5 min before adding 15 μl of an equine serum sample. Further, subsequent steps of incubation of serum samples and conjugate were conducted for 45 min each. The anti-horse enzyme-conjugate came as a ready-to use component. Equal to LIA B, the second washing process with WB (3 x 5 min. each) was followed by immediate application of the substrate and its incubation for 10 min. To stop the color reaction, fluids were poured off and strips were rinsed with 1.5 ml *Aq*. *dest*. three times without incubation. After finishing the test, air-drying the strips for at least 20 min away from sunlight and attaching them to an evaluation sheet, evaluation was performed using a flatbed scanner and evaluation software (version 6.0.0) supplied by the manufacturer.

Color reactions on single AG lines were evaluated and categorized automatically by the software with values from 0 to 0.9 (negative (neg); no signal or signal fainter than COC), 1.0 (equivocal positive (equivoc); signal equal to COC), and greater than 1.0 (positive (pos); strong signal greater than COC). The overall rating of an entire test strip or serum sample in LIA C and its assignment to an overall test result allocation category is explained in Table 4. Hereby, the manufacturer of LIA C distinguishes between allocation categories “NEGATIVE (NEG)–no evidence of relevant contact with pathogen”, “EQUIVOCAL (EQUIVOC)–evidence of contact with pathogen”, “POSITIVE (POS) (infection)–evidence of infection“, “VACCINATED (VAC)–evidence of vaccination”, and “VACCINATED AND POSITIVE (VAC+POS)–evidence of vaccination and infection”. The OspA AG signal was considered as specific AG signal in equine immunoreactions and considered accordingly in the evaluation of a test.

**Table 4:** LIA C–overall rating of a test strip or equine serum sample and its assignment to an overall test result allocation category according to manufacturers’ instructions.

Allocation categories (overall test result)	Antigen line evaluation—Equine sera
**NEG**	0 AG lines or AG lines < COC or 0–2 AG lines ≥ COC (except OspA or VlsE / C6 AG line)
**EQUIVOC**	VlsE / C6 AG line and 0–2 AG lines ≥ COC (except OspA or p18 AG line) or 3 AG lines ≥ COC (except OspA or VlsE / C6 AG line)
**POS**	VlsE / C6 AG line and ≥ 3 AG lines ≥ COC (except OspA or p18 AG line) or ≥ 4 AG lines ≥ COC (except OspA or VlsE / C6 AG line) or VlsE / C6 AG line, p18 AG line and ≥ 1 AG line ≥ COC (except OspA AG line)
**VAC**	OspA AG line and ≥ 0–2 AG lines ≥ COC or OspA AG line and 0–3 AG lines ≥ COC (except VlsE / C6 AG line)
**VAC+POS**	OspA AG line, VlsE / C6 AG line and ≥ 3 AG lines ≥ COC (except p18 AG line) or OspA AG line and ≥ 4 AG lines ≥ COC (except VlsE / C6 AG line) or OspA AG line, VlsE / C6 AG line, p18 AG line and ≥ 1 AG lines ≥ COC

Overall rating and allocation are based on the AG line results obtained with LIA C and its evaluation according to LIA C’s evaluation protocol for horses supplied by the manufacturer. VlsE and C6 were considered as one AG line albeit both AG lines might appear ≥ cut off control AG line (COC). The appearance of one of those two lines alone was **not** sufficient to allocate samples to the categories “EQUIVOC”, “POS” as well as “VAC+POS”.

AG line, antigen line; NEG, negative overall test result; EQUIVOC, equivocal overall test result; POS, positive overall test result (infection); VAC, vaccinated overall test result (vaccination); VAC+POS, vaccinated and positive overall test result (vaccination and infection); COC, cut off control; OspA, outer surface protein A; VlsE, variable major protein-like sequence expressed; C6, peptide referring to 6^th^ invariant region of VlsE; p18, protein 18 (synonymous with decorine binding protein A (DbpA)).

### 2.3. Statistical methods

For each serum sample and LIA strip the signal intensity results on single *Bb*sl-specific antigen lines and the overall test result were recorded, organized, and analyzed with Microsoft Excel Office 365 (Microsoft Corporation, Redmond, WA, USA). Results were grouped and charted by experimental group, LIA and sampling time-point (d0, d135 and d210).

To assess the agreement of results between the three independent raters (named LIA A, LIA B and LIA C), single AG-AB immunocomplex signal intensity results and overall test results were statistically analyzed for each serum sample using the Fleiss’ kappa test [24, 25]. The observed inter-rater agreement (P_o_) and the statistic inter-rater reliability (IRR), represented by the Fleiss´ kappa coefficient (*κ*), were calculated per sampling time-point; once without and second with additional subdivision into experimental groups (Non-Vac, Vac-Basic, Vac-Plus). The IRR was categorized into “poor” (*κ* = < 0.0), “slight” (*κ* = 0.0 to 0.2), “fair” (*κ* = 0.21 to 0.40), “moderate” (*κ* = 0.41 to 0.60), “substantial” (*κ* = 0.61 to 0.80), and “almost perfect” (*κ* = 0.81 to 1.00). A high value for *κ* represents a high agreement of the three tests or raters regarding a sample`s overall result, or its coloration of single AG signals respectively [26].

## 3. Results

The study was conducted with, in total 393 equine sera of 131 clinically healthy horses priorly diagnosed as LB-negative or equivocal with an in-house KELA and a commercial LIA. Each horse was assigned to one of three experimental groups–non-vaccinated group Non-Vac (*n =* 42), which served as negative control group or vaccinated group Vac-Basic (*n =* 45) or Vac-Plus (*n =* 44) (S1 Table). Serum samples collected at three different sampling time-points in the vaccination schedule (d0, d135 and d210) were analyzed for their antibody contents each with LIA A, LIA B and LIA C.

### 3.1. Results after evaluation and allocation according to manufacturers’ instructions

All three LIAs were tested with 393 equine serum samples each and handling was without any issues. Visual evaluation of test strips in LIA A, and automatic scan and interpretation of results on test strips in LIA B was entirely successful. However, for LIA C the automatic scan and evaluation was not successful for three strips incubated with serum samples from one and the same horse at three time-points (ZP0-28, ZP3-28, ZP4-28) as its COCs were above the maximum level the scanner was able to process. Subsequently, the evaluation was performed visually by the examiner in this case.

#### 3.1.1. Results pertaining to specific antigen signals

Results for single AG lines of every LIA strip and serum sample were analyzed by signal intensity and recorded by experimental group, sampling time-point and LIA. To contrast results between LIAs, the number of samples with a signal intensity result of “no signal“, “signal intensity fainter than COC“, “signal intensity equal to COC”and “signal intensity greater than COC”was calculated for each *Borrelia*-specific AG tested. Results were charted by experimental group, sampling time-point and LIA. Additionally, the observed inter-rater agreement (P_o_) and IRR (*κ*) were calculated for signal intensity results of single antigens tested. Special attention was put on results for vaccination-specific OspA AG signal, as well as VlsE AG signal as the leading parameter indicating an infection with pathogens of the *Bb*sl-complex.

**Fig 1:**
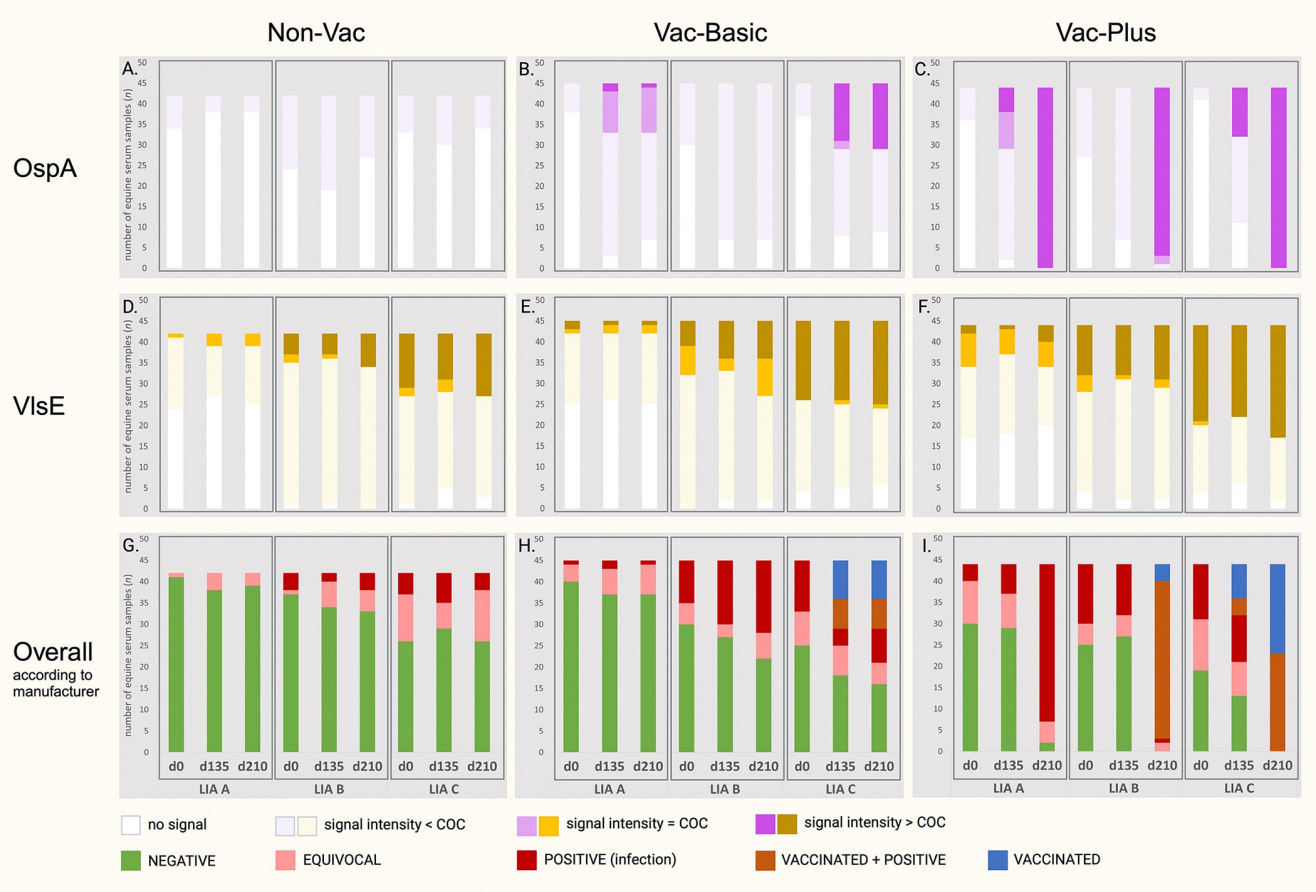
**(A)-(I).** Comparison of test results obtained by three LIAs used for *Bb*sl-serodiagnosis with equine serum samples. Evaluation was performed according to the three manufacturers’ instruction manuals. Test results are charted by experimental group (Non-Vac (*n* = 42), Vac-Basic (*n* = 45), Vac-Plus (*n* = 44)), LIA (LIA A, LIA B and LIA C) and time-point of blood sample collection (d0, d135, d210). **(A)-(C).** Comparison of OspA AG-AB immunocomplex signal intensity results. The number of serum samples with a “negative”OspA signal intensity result are displayed in white (no signal) and lilac (signal fainter than COC), the number of samples with a “positive”OspA signal intensity result are illustrated in light purple (signal equal to COC) and bright purple (signal greater than COC). **(D)-(F).** Comparison of VlsE AG-AB immunocomplex signal intensity results. The number of serum samples with a “negative”VlsE signal intensity result are represented by white color (no signal) and vanilla (signal fainter than COC), number of samples with a “positive”VlsE signal intensity result are displayed in yellow (signal equal to COC) and ocher (signal greater than COC). **(G)-(I).** Comparison of overall test results. The number of serum samples with an overall test result of “NEGATIVE” (green), “EQUIVOCAL”(salmon), “POSITIVE (infection)”(red), “VACCINATED AND POSITIVE” (light brown) and “VACCINATED” (blue) are illustrated. In LIA A, vaccination-specific OspA is not considered as evidence for immunization and, therefore, there is no allocation in terms of “VACCINATED” and “VACCINATED AND POSITIVE” in this test. *Bb*sl, *Borrelia burgdorferi* sensu lato; Non-Vac, non-vaccinated horses; Vac-Basic, horses vaccinated on d0 and d14; Vac-Plus, horses vaccinated on d0, d14 and d180; d, day of blood sample collection during the vaccination schedule; AG, antigen; AB, antibody; OspA, outer surface protein A; VlsE, variable major protein-like sequence expressed; COC, cut off control.

*A*. *OspA antigen signal*. None of the three LIAs displayed “positive”OspA signal intensity results (equal to or greater than the COC) for samples of group Non-Vac at any time-point in the observational period. In other words, there was no color reaction signal on the OspA AG line or it was fainter than the COC and, therefore, was considered as “negative” for all samples of group Non-Vac at d0, d135 and d210 (Fig 1A–1C). Likewise, none of the three LIAs displayed a positive OspA AG signal result at d0 for none of the samples in the vaccinated groups Vac-Basic and Vac-Plus.

The observed inter-rater agreement (P_o_) for OspA signal intensity results at d0 ranged from 69% to 63% depending on group, the calculated IRR (κ) was slight for all three groups at d0 (S3 Table). For group Non-Vac at d135 the P_o_ was 56% while the kappa was poor (κ = -0.04), yet, it increased at d210 (P_o_ = 70%, κ = 0.10 (slight). In LIA A at d135, the antibody level of only a fraction of vaccinated horses of groups Vac-Basic and Vac-Plus was sufficient to induce a positive result for OspA AG signal (*n*_Vac-Basic_ = 12/45, *n*_Vac-Plus_ = 15/44). Similarly, at d210 and group Vac-Basic only 27% of the vaccinated horses (*n*_Vac-Basic_ = 12/45) got identified as such by LIA A; yet, in group Vac-Plus OspA-specific AB got detected with highly positive signal intensity results in 100% of the samples (*n*_Vac-Plus_ = 44/44).

In LIA B at d135, none of the vaccinated horses displayed a positive result for OspA at d135 (*n*_Vac-Basic_ = 0/45, *n*_Vac-Plus_ = 0/44). At d210, repeatedly none of the basic-vaccinated horses was identified as such by LIA B (*n*_Vac-Basic_ = 0/45); in group Vac-Plus, though, LIA B positively detected OspA-specific AB in 98% of the samples (*n*_Vac-Plus_ = 43/44).

In LIA C, the antibody level was sufficient to induce a positive OspA signal in 36% of the samples of group Vac-Basic (*n*_Vac-Basic_ = 16/45) and 27% of the samples of group Vac-Plus (*n*_Vac-Plus_ = 12/44) at d135, and, in 36% of the samples of group Vac-Basic (*n*_Vac-Basic_ = 16/45) at d210. In group Vac-Plus, LIA C identified all vaccinated horses by highly positive OspA signal intensity results in 100% of the samples (*n*_Vac-Plus_ = 44/44).

In summary, at d135 only LIA A and LIA C positively detected OspA-specific antibodies in samples of the two vaccinated groups, but not LIA B. At d210 and group Vac-Basic, again, only LIA A and LIA C did so, but not LIA B; yet, in group Vac-Plus at d210 all three LIAs displayed highly positive results for OspA AG signal. In general, LIA C reacted the most sensitive–closely followed by LIA A–by displaying the highest number of samples with an OspA signal intensity result greater than the COC, while LIA B clearly demonstrated the lowest sensitivity in regard to OspA-specific AB. Alike for group Non-Vac at d135, the observed inter-rater agreement in regard to OspA AG signal results dropped to a low for groups Vac-Basic (P_o_ = 50%) and Vac-Plus (P_o_ = 53%), too, at a slight IRR. At d210, P_o_ significantly increased again to a high (P_o_ = 61% for group Vac-Basic, P_o_ = 95% for group Vac-Plus). Nevertheless, the calculated IRR at d210 dropped to poor for group Vac-Plus (κ = -0.02), while in group Vac-Basic it increased to fair (κ = 0.30).

*B*. *VlsE antigen signal*. When comparing results of LIA A, LIA B and LIA C in regard to signal intensity results for VlsE AG signal, LIA C generally reacted the strongest followed by LIA B and LIA A.

LIA A displayed the lowest number of VlsE-positive samples (signal intensity result ≥ COC) at all three time-points and groups (d0: *n*_Non-Vac_ = 1/42, *n*_Vac-Basic_ = 3/45, *n*_Vac-Plus_ = 10/44; d135: *n*_Non-Vac_ = 3/42, *n*_Vac-Basic_ = 3/45, *n*_Vac-Plus_ = 7/44; d210: *n*_Non-Vac_ = 3/42, *n*_Vac-Basic_ = 3/45, *n*_Vac-Plus_ = 10/44), yet, overall with a high in group Vac-Plus.

LIA B showed a higher number of VlsE-positive samples in comparison to LIA A in all three groups at three time-points (d0: *n*_Non-Vac_ = 7/42, *n*_Vac-Basic_ = 13/45, *n*_Vac-Plus_ = 16/44; d135: *n*_Non-Vac_ = 6/42, *n*_Vac-Basic_ = 12/45, *n*_Vac-Plus_ = 13/44; d210: *n*_Non-Vac_ = 8/42, *n*_Vac-Basic_ = 18/45, *n*_Vac-Plus_ = 15/44), yet, overall with a low in group Non-Vac.

LIA C presented by far the most VlsE-positive samples in all three groups at three time-points (d0: *n*_Non-Vac_ = 15/42, *n*_Vac-Basic_ = 19/45, *n*_Vac-Plus_ = 24/44; d135: *n*_Non-Vac_ = 14/42, *n*_Vac-Basic_ = 20/45, *n*_Vac-Plus_ = 22/44; d210: *n*_Non-Vac_ = 15/42, *n*_Vac-Basic_ = 21/45, *n*_Vac-Plus_ = 27/44).

Independent of the experimental group and LIA, there was no significant increase apparent in the number of VlsE-positive samples over the progressing sampling time-points in the vaccination schedule (Fig 1D–1F). In general, all three LIAs detected VlsE-specific AB (≥ COC) most frequently in serum samples of horses belonging to group Vac-Plus–independent of the time-point of blood sample collection.

In summary, LIA C reacted very sensitive for VlsE-specific antibodies with up to 61% of VlsE-positive samples in group Vac-Plus, while LIA A clearly was least sensitive in this aspect. In consequence, there´s a high discrepancy between the three LIAs regarding the detection of VlsE-specific antibodies. Accordingly, the calculated IRR was poor to slight at d0 at an observed inter-rater agreement of 38% in group Non-Vac, 36% in group Vac-Basic and 39% in group Vac-Plus. At d135, the P_o_ and IRR mostly remained unchanged for all three groups (Non-Vac: P_o_ = 31%, κ =

-0.15; Vac-Basic: P_o_ = 33%, κ = -0.05; Vac-Plus: P_o_ = 40%, κ = 0.09). At d210, the poor agreement of results between the three LIAs did not improve either (Non-Vac: P_o_ = 34%, κ = -0.09; Vac-Basic: P_o_ = 31%, κ = -0.07; Vac-Plus: P_o_ = 32%, κ = -0.01) (S3 Table).

#### 3.1.2. Overall test results

The number of samples with an overall evaluation result of “NEGATIVE” (NEG), “EQUIVOCAL” (EQUIVOC), “POSITIVE” (POS), “VACCINATED AND POSITIVE” (VAC+POS) as well as “VACCINATED” (VAC) was calculated and charted by experimental group, LIA and sampling time-point. Overall, LIA C reacted the strongest at all three time-points and groups, followed by LIA B and LIA A (Fig 1G–1I).

At d0 and d135, LIA A displayed the highest number of “NEG” samples in all three groups, as well as at d210 in groups Non-Vac and Vac-Basic. In contrast, in group Vac-Plus at d210, LIA A assessed the greatest number of samples as POS in comparison to LIA B and LIA C due to the fact that OspA was not considered as vaccination-specific and, consequently, the absence of allocation categories pertaining to vaccinated or vaccinated and infected horses in LIA A’s technical manual. Therefore, at d210 none of the samples of vaccinated horses were allocated as VAC, or VAC+POS, which resulted in many false-positive overall test results in vaccinated groups in LIA A.

In comparison to LIA A, more sensitive LIA B rated a higher number of samples as POS and a lower number of samples as NEG in all three groups at d0 and d135, as well as in groups Non-Vac and Vac-Basic at d210. At d210, LIA B identified 84% of the vaccinees in group Vac-Plus as VAC+POS (*n* = 37/44) while only four samples were allocated as VAC.

In contrast, most sensitive LIA C showed the lowest number of “NEG” samples at three time-points and groups. Yet, based on the detection of OspA-specific antibodies, LIA C detected the highest number of vaccinees at d135 and d210 in the two vaccinated groups. Here, in comparison to LIA B, LIA C allocated a significantly lower number of horses as VAC+POS (*n* = 23/44) and, instead, more horses as VAC (*n* = 21/44).

For the overall test results of 131 equine sera at three classified time-points (d0, d135, d210)–without subdivision of results into groups–the observed inter-rater agreement (P_o_) and the statistic inter-rater reliability (IRR, *κ*) were 66% and fair (*κ* = 0.28) at d0, 55% and slight (*κ* = 0.18) at d135, as well as 41% and slight (*κ* = 0.18) at d210. With additional classification of results into experimental groups, the IRR did not improve despite a partly increased observed inter-rater agreement. That is, group Non-Vac (*n* = 42) displayed a slight IRR at all three time-points, yet, in combination with the highest observed inter-rater agreement (d0: P_o_ = 71%, d135: P_o_ = 70%, d210: P_o_ = 63%) (S4 Table). For group Vac-Basic (*n =* 45) the IRR was fair at d0 (*κ* = 0.28, P_o_ = 67%), slight at d135 (*κ* = 0.15, P_o_ = 50%), and slight at d210 (*κ* = 0.11, P_o_ = 44%), too. The highest discrepancy in the accordance of overall results between LIAs was observed in group Vac-Plus (*n =* 44), in particular in allocation categories “POS”, “VAC” and “VAC+POS”; for group Vac-Plus the IRR was fair at d0 (*κ* = 0.32, P_o_ = 60%), dropped to slight at d135 (*κ* = 0.18, P_o_ = 47%) and further dropped to poor (*κ* = -0.23) at d210 at a low of P_o_ of 17%.

In summary, LIA C displayed the highest sensitivity, particularly in terms of the detection of VlsE-specific AB, while it was equally sensitive to LIA A regarding the detection of OspA-specific AB. LIA B showed the lowest sensitivity towards OspA-specific AB, while LIA A did so in regard to VlsE-specific AB. In consequence, LIA C demonstrated the highest specificity at the identification of vaccinated horses, followed by LIA A. In this context, LIA B demonstrated the lowest specificity.

### 3.2. Results after evaluation and allocation according to an alternative recommended overall evaluation protocol (ROEP) for LIAs and equine serum samples

In addition to the evaluation of LIA strips according to manufacturers’ evaluation protocols all LIA strips in this study were reevaluated with an alternative, uniform and recommended overall evaluation protocol (ROEP) for equine serum samples. This protocol is in accordance with instruction manuals for sera of vaccinated and vaccinated and infected dogs. It was established to counteract shortcomings of non-standardized LIA evaluation protocols for equine sera supplied by manufacturers, and to refine and standardize the interpretation of LIA results in order to improve serodiagnostic accuracy for equine LB.

The application of ROEP solely had influence on the overall test results, that is, evaluation of single AG-AB immunocomplex signal results remained unchanged and was performed according to the respective manual or computer software of a LIA. In the overall evaluation process ROEP considered the OspA signal as vaccination-specific in all three LIAs. In this context, the appearance of positive color reaction signals (≥ COC) on additional antigen lines–except VlsE–that are known to occur in LIA of vaccinated dogs, are considered as reaction to the various antigens provided with the lysate vaccine applied, respectively.

According to the ROEP a differentiation between the five allocation categories “NEGATIVE” (NEG), “EQUIVOCAL” (EQUIVOC), “POSITIVE (infection)” (POS), “VACCINATED” (VAC) and “VACCINATED AND POSITIVE” (VAC+POS) was performed (Table 5).

**Table 5:** Recommended overall evaluation protocol (ROEP)–overall rating of a test strip or equine serum sample and its assignment to an overall test result allocation category according the ROEP.

Allocation categories (overall test result)	Antigen signal evaluation—Equine sera
**NEG**	0 AG signals or AG signals < COC or 0–2 AG signals ≥ COC (except OspA or VlsE / C6* AG signal)
**EQUIVOC**	VlsE / C6* AG signal and 0–2 AG signals ≥ COC (except OspA or DbpA** AG signal) or VlsE / C6* AG signal, DbpA** AG signal and 0 AG signals ≥ COC or VlsE / C6* AG signal, OspA AG signal and 2 AG signals ≥ COC (except DbpA** AG signal) or 3 AG signals ≥ COC (except OspA or VlsE / C6* AG signal)
**POS**	VlsE / C6* AG signal and ≥ 3 AG signals ≥ COC (except OspA** AG signal) or ≥ 4 AG signals ≥ COC (except OspA or VlsE / C6* AG signal) or VlsE / C6* AG signal, DbpA** AG signal and ≥ 1 AG signal ≥ COC (except OspA AG signal)
**VAC**	OspA AG signal and ≥ 0–2 AG signals ≥ COC or OspA AG signal and 0–5 AG signals ≥ COC (except VlsE / C6* AG signal)
**VAC+POS**	OspA AG signal, VlsE / C6* AG signal and ≥ 3 AG signals ≥ COC or OspA AG signal, VlsE / C6* AG signal, DbpA** AG signal and ≥ 1 AG signals ≥ COC

Overall rating and allocation are based on the AG-AB immunocomplex signal intensity results obtained with LIA and its evaluation according to an alternative ROEP for equine serum samples. VlsE and C6 (*if present) are considered as one AG signal albeit both signals might appear equal to or greater than the cut off control AG signal (COC). **DbpA protein is considered as synonymous with p18 protein.

AG, antigen; AB, antibody; NEG, negative overall test result; EQUIVOC, equivocal overall test result; POS, positive overall test result (infection); VAC, vaccinated overall test result (vaccination); VAC+POS, vaccinated and positive overall test result (vaccination and infection); COC, cut off control; OspA, outer surface protein A; VlsE, variable major protein-like sequence expressed; C6, peptide referring to 6^th^ invariant region of VlsE; DbpA, decorine binding protein A.

#### 3.2.1. Overall test results

Reassessed, the number of samples with an overall evaluation and allocation result of “NEGATIVE” (NEG), “EQUIVOCAL” (EQUIVOC), “POSITIVE (infection)” (POS), “VACCINATED AND POSITIVE” (VAC+POS) as well as “VACCINATED” (VAC) was calculated and charted by experimental group, LIA and sampling time-point.

**Fig 2:**
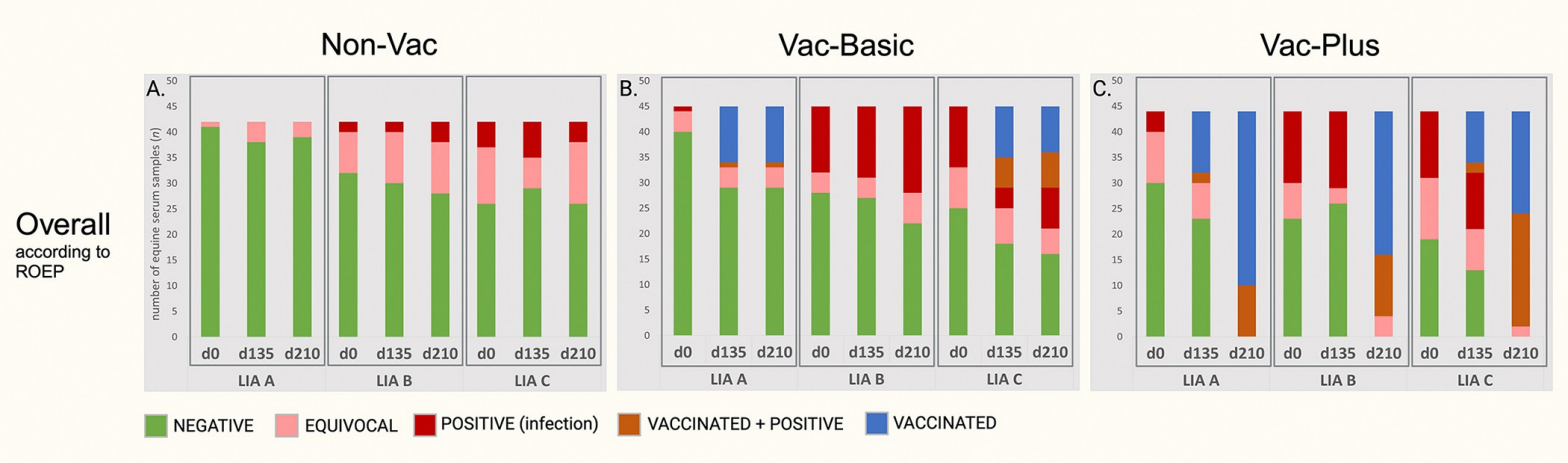
**(A)-(C)**. Overall test results after application of the ROEP–comparison of overall test results obtained by three LIAs used for LB serodiagnosis in horses. Evaluation and allocation were performed according to an alternative recommended overall evaluation protocol for equine serum samples which considered OspA signal as specific for equine immunoreaction. The number of serum samples with an overall test result of “NEGATIVE” (green), “EQUIVOCAL”(salmon), “POSITIVE”(red), “VACCINATED AND POSITIVE” (light brown) and “VACCINATED” (blue) after evaluation with the ROEP are illustrated. Results are charted by experimental group (Non-Vac (n = 42), Vac-Basic (n = 45), Vac-Plus (n = 44)), LIA (LIA A, LIA B, LIA C) and time-point of blood sample collection (d0, d135 and d210). ROEP, recommended overall evaluation protocol; d, day of blood sample collection during the vaccination schedule; Non-Vac, non-vaccinated horses; Vac-Basic, horses vaccinated on d0 and d14; Vac-Plus, horses vaccinated on d0, d14 and d180.

After application of ROEP, in LIA A there is neither a divergence in test results in group Non-Vac at no point, nor in groups Vac-Basic and Vac-Plus at d0. Yet, the effect of ROEP becomes apparent in the two vaccinated groups at d135 and d210: the former number of “NEG” and “EQUIVOC” samples slightly decreased, the number of “POS” samples decreased to none, and the samples recognized as vaccinated significantly increased (VAC_d135_: *n*_Vac-Basic_ = 11/45, *n*_Vac-Plus_ = 12/44; VAC_d210_: *n*_Vac-Basic_ = 11/45, *n*_Vac-Plus_ = 34/44; VAC+POS_d135_: *n*_Vac-Basic_ = 1/45, *n*_Vac-Plus_ = 2/44; VAC+POS_d210_: *n*_Vac-Basic_ = 1/45, *n*_Vac-Plus_ = 10/44), since the immunization-induced positive OspA signal is now assessed as specific for vaccination (Fig 2A–2C).

In LIA B, similarly, the application of ROEP resulted in a significant increase in the number of “VAC” horses and a decrease in the amount of “VAC+POS” horses, yet, only in group Vac-Plus at d210. That is, formerly 9% of “Vac-Plus” samples were allocated as VAC (*n* = 4/44) and 84% as VAC+POS (*n* = 37/44), while with application of ROEP 64% of “Vac-Plus” samples were then allocated as VAC (*n* = 28/44) and 25% as VAC+POS (*n* = 12/44) at d210. In group Vac-Plus at d0 and d135, as well as in group Vac-Basic at all three time-points, there`s no significant alteration in overall allocation results of samples since the alternate overall evaluation protocol has no influence on the detection rate of OspA-specific AB in LIA B. Also, in the non-vaccinated group the application of ROEP produced divergent overall test results in LIA B (S5 Table). It resulted in an increase of “EQUIVOC” samples combined with a decrease of “NEG” samples at three time-points; at d0, five samples’ allocation result changed from NEG to EQUIVOC and two from POS to EQUIVOC, at d135 four got altered NEG to EQUIVOC, one from POS to EQUIVOC and one from EQUIVOC to POS, and at d210 one sample shifted from POS to EQUIVOC, one from POS to EQUIVOC and one sample from EQUIVOC to POS.

In LIA C there is no alteration in the overall evaluation and allocation results neither in group Non-Vac at any time-point, nor in the vaccinated group at d0. In the groups of vaccinated horses at d135 and d210, there is no significant disagreement in results after application of ROEP; at d135, one sample`s allocation result in group Vac-Basic changed from VAC+POS to VAC, while in group Vac-Plus two samples moved from VAC+POS to VAC. At d210, two samples`overall result in group Vac-Plus changed from VAC to EQUIVOC, and one changed from VAC+POS to VAC.

In view of the inter-rater agreement, the calculated IRR (*κ*) partially improved after application of ROEP. When comparing the inter-rater agreement results after application of manufacturers`evaluation protocols with those after application of the ROEP, the IRR for overall results for 131 equine serum samples–without subdivision into experimental groups–at first, remained fair at d0 (*κ* = 0.35, P_o_ = 68%), yet, increased at d135 (*κ* = 0.24 (fair), P_o_ = 54%) and d210 (*κ* = 0.43 (moderate), P_o_ = 59%) with application of ROEP (S4 Table).

With additional subdivision of results into experimental groups, the agreement of results in group Non-Vac (*n =* 42) increased at all three time-points (d0: *κ* = 0.22 (fair), P_o_ = 72%; d135: *κ* = 0.22 (fair), P_o_ = 71%; d210: *κ* = 0.17 (slight), P_o_ = 66%). For group Vac-Basic (*n =* 45) the inter-rater agreement negligibly improved at all three time-points (d0: *κ* = 0.33 (fair), P_o_ = 68%; d135: *κ* = 0.19 (slight), P_o_ = 48%; d210: *κ* = 0.18 (slight), P_o_ = 44%). The assets of ROEP mainly become apparent in group Vac-Plus (*n =* 44): while the agreement between raters remained fair at d0 (*κ* = 0.38, P_o_ = 63%) and d135 (*κ* = 0.20, P_o_ = 45%), it considerably climbed from poor to fair at d210 (*κ* = 0.33, P_o_ = 67%).

## 4. Discussion

An increased risk of transmission and subsequent infection with members of the *Borrelia burgdorferi* sensu lato complex is expected due to an increase in tick activity and georgical spread. Following the infection with organisms of the *Bb*sl-complex the host responds with the production of specific antibodies against the infectious agent. Some individuals may also develop clinical signs. Consequently, infection and clinical disease are two different entities. Seropositivity itself is an indicator for a past or active exposure to borreliae antigens, yet, not necessarily associated with clinical disease. Although there are clinical case reports, in which an association between antibody positivity and disease in horses was suggested, *Bb*sl infection in animals often remains clinically inapparent or clinical signs are vague [27–33]. In consequence, only horses with occurrence of clinical signs delineated for a LB (i. a. intermittent lameness, encephalitis, uveitis, infectious arthritis, cutaneous pseudolymphoma [8–10]), supported by a positive serodiagnostic test result should receive antibiotic treatment.

Thereby, it is essential to have highly sensitive and specific serodiagnostic tools available that produce accurate, reliable and reproduceable test results to confirm a *Bb*sl infection in order to support the suspected diagnosis. Misdiagnosis or misclassification of a diagnostic outcome impedes clinical decision-making and potentially prevents appropriate treatment or results in mistreatment of the patient. A false-negative diagnostic outcome on the one hand may lead to an undiscovered or late detected infection and the omission of appropriate on-time antibiotic treatment potentially causing persisting clinical signs. A false-positive test result on the other hand is just as problematical due to unnecessary antibiotic treatment with its detrimental effects on the horses’ health (i. a. effects of drug intolerance, diarrhea due to colitis, increased risk of additional infections) and the risk of antibiotic-resistance development in bystander bacteria [16].

In regard to LIAs used for the detection and confirmation of equine LB, it should feature a suitable sensitivity and a high specificity to be able to accurately detect *Bb*sl-specific AB. Subsequently, the diagnostic outcome must be adequately interpreted and classified according to a LIA evaluation protocol for equine sera validated and standardized across various tests. Here, the differentiation of a vaccination-induced immune response from those initiated by a past or active infection due to natural exposure must be performed by interpreting vaccination- and infection-specific markers (i. e. OspA and VlsE/ C6 AG line) appropriately. In addition, a LIA should be easy to apply and offer a user-friendly operability.

To make headway in the challenging diagnosis of LB in horses with non-standardized serodiagnostic tests, we qualitatively assessed three LIAs available for serodiagnosis of LB in equine serum samples. Tests were evaluated and compared regarding their sensitivity, specificity, diagnostic outcome and the operability of a test using serum samples of non-vaccinated and vaccinated horses. Herby, special attention was put on the sensitivity and specificity of vaccination-specific OspA AG line and infection-specific VlsE AG line. In addition, AG signals of all three LIAs where again interpreted and classified using a uniform recommended overall evaluation protocol (ROEP) with the aim to refine and standardize serodiagnosis of equine LB across LIAs available in this study.

### 4.1. Sensitivity, specificity, and overall results

In terms of identifying non-vaccinated and vaccinated horses, the three assessed tests varied in their accuracy in detecting vaccination-induced specific antibodies against OspA AG. Thereby, the inter-rater-agreement for OspA AG signal intensity results and overall test results ranged from fair to poor depending on time-point of blood sample collection and group, representing an unsatisfying agreement of results of the three LIAs in this aspect.

Generally, serum samples of non-vaccinated horses, as well as samples of horses from groups Vac-Basic and Vac-Plus at d0, which had received their first vaccination after the first blood sample collection, should not display a positive OspA color reaction signal at any time-point, since OspA AG is not expressed by active borreliae during an ongoing infection. In contrast, LB inactivated lysate vaccines available for horses contain OspA, thus, specific antibodies against OspA AG in serum are a consistent marker for immunization against LB. At sampling time-point d135, 121 days after the second immunization of group Vac-Basic and Vac-Plus, and at sampling time-point d210, 196 days after the last immunization of group Vac-Basic and 30 days after the third immunization of group Vac-Plus, a vaccination-specific positive OspA signal is expected for horses belonging to these two groups. Taking the humoral immune response of horses after immunization with commercial vaccines against LB into account, the OspA signal intensity result at d210 is expected to be stronger for samples of group Vac-Plus than those of Vac-Basic due to a more recent vaccination and, therefore, a higher antibody level in horses of group Vac-Plus [12]. It also needs to be considered that the application of lysate vaccines against LB in horses may result in the development of antibodies against a variety of other borreliae proteins that are expressed by borreliae in *vitro* (i. e., OspC, DbpA, BmpA, p58, or p100). That is, the appearance of respective immunocomplex reaction signals on LIA strips in combination with a positive reaction to OspA AG can occur in vaccinated animals. Thus, it should not be counted as infection-specific, yet be considered as vaccination-induced, and evaluation and allocation should be exercised accordingly. Only VlsE and C6 AG signals are highly specific markers for a *Bb*sl-infection, since those are only expressed by active borreliae in *vivo* [21].

Correspondingly to expected OspA-specific AB reactions in non-vaccinated horses, none of the three LIAs displayed a signal result for OspA equal to or greater than COC for none of the samples of non-vaccinated horses at any time-point. None of the three LIAs falsely identified non-vaccinated horses (horses of group Non-Vac at d0, d135, d210 and groups Vac-Basic and Vac-Plus at d0) as vaccinated. Nevertheless, the observed inter-rater agreement and IRR for OspA AG signal in this group was not greater than 70% and slight (κ ≤ 0,10) at no point. Despite uniformly negatively rated OspA AG signal results for non-vaccinated horses, the sensitivity and specificity of tests in regard to OspA and, therefore, signal intensity results within the below-COC-range significantly varied between LIAs resulting in a low agreement of tests. While LIA A and LIA C demonstrated a high specificity in non-vaccinated horses in this context, LIA B by far displayed the lowest.

In contrast to a correct identification of non-vaccinated horses, none of the three tests correctly identified all vaccinated horses (Vac-Basic and Vac-Plus, *n* = 89). LIA B completely failed to positively detect OspA-specific AB in serum of all vaccinated horses at d135 and all Vac-Basic horses at d210, revealing a severe lack of sensitivity and specificity in this aspect. Although LIA A and LIA C demonstrated a significantly higher sensitivity and specificity towards OspA-specific AB, it was not satisfying in regard to Vac-Basic horses, either.

Next to a lack of sensitivity of tests, an additional explanation for the limited OspA detection rate in Vac-Basic horses may be an insufficient OspA AB titer in the vaccinated horses’ blood samples. Besides an appropriate sensitivity of AG signals on a test, a sufficient OspA AB titer in vaccinated horses is essential for the correct detection of LB in horses and a successful differentiation from a vaccination, respectively. In this context, the high OspA detection rate in additionally vaccinated horses at d210 demonstrates that a gap in the vaccination schedule is disadvantageous while a tight vaccination schedule and a sufficient vaccination-induced AB titer (especially OspA-specific AB) are highly beneficial. On the one hand, it enables the highest possible protective effect against natural LB infection and disease. On the other hand, it facilitates the identification of vaccinated animals via OspA-specific AB, and their differentiation from naturally infected animals. In other words, a sufficient OspA AB titer against *Bb*sl indicates a general vaccination-induced immunoreaction allowing a correct demarcation of vaccinated horses against naturally infected horses, and thus, limits the risk of possibly false-positive overall test results for vaccinated horses and its side effects.

Another issue concerning the correct identification of vaccinated horses was the interpretation of single AG lines and its overall evaluation and allocation in both, LIA A and LIA B.

In LIA A´s evaluation manual supplied by the manufacturer, the OspA AG line was not at all considered as specific AG line in equine immunoreactions. Correspondingly, no allocation categories pertaining to a vaccination or both, a vaccination and an infection, were included in LIA A’s basic evaluation manual. In consequence, despite that LIA A was technically capable of detecting OspA-specific AB at a high extent and simultaneously demonstrated a high sensitivity and reliable specificity in regard to OspA-specific AB, the positive OspA AG line results were not interpreted appropriately, resulting in many false-positive overall test results for vaccinated horses.

Similarly, in LIA B ´s evaluation manual supplied by the manufacturer–despite its consideration of the OspA AG line as vaccination-specific–the interpretation and allocation of single AG lines was fragmentary, too, resulting in misinterpretation of results of vaccinated horses. In comparison to the well-thought-out evaluation manual of LIA C, LIA B ´s technical manual did not further specify the case of a vaccination combined with an infection, thus, did not include an allocation category in the manual pertaining to this case. However, in the software respective samples were allocated to an additional allocation category termed “Positive–serological evidence of infection *or* immunization”. Being aware of the incorrect specification and terminology, pertinent samples were allocated to LIA C`s VAC+POS allocation category in the statistical analysis performed. In this context, there was also no further specification regarding the interpretation of AG lines such as infection-specific VlsE AG line, clearly demonstrating a deficit in the evaluation and allocation protocol of LIA B, as well as a dubiety of the test´s value pertaining to overall results for vaccinated horses.

Despite that the application of the alternative consistent evaluation protocol for equine serum samples as recommended above (ROEP) cannot compensate for different sensitivities of antigen-coats on LIA strips of the different manufacturers. The consistent evaluation protocol improved inter-rater agreement in group Vac-Plus at d210, when all three LIAs detected OspA-specific AB to an equal extent and subsequent interpretation of AG lines was exercised alike.

Regarding the identification of naturally infected horses (POS), or vaccinated and infected (VAC+POS), the VlsE (and C6) AG signal should be considered and evaluated as highly specific marker for a *Bb*sl-infection [21, 22]. Hereby, it is important that the sensitivity of a test´s VlsE AG signal is sufficient to reliably detect VlsE-specific AB. Yet, its specificity in this aspect is from paramount importance since a positive VlsE AG signal result–in combination with possibly further positive AG lines (except OspA)–is highly indicative for an ongoing infection and the necessity of a prompt antibiotic treatment of the *Bb*sl-infected horse with clinical signs. Similarly, a too high sensitivity as well as a lack of specificity of the VlsE AG signal are equally precarious since it might entail a false-positive overall test result. Here, it needs to be deliberated on whether it is beneficial to have a very high sensitivity of VlsE AG signal on test strips and, in consequence, potentially a lack of specificity and a questionable significance of a test´s “POS” overall test result. A down-regulation of sensitivity for VlsE AG signals might be advisable; that is, in case of doubt of an EQUIVOC test result determined by a less sensitive VlsE AG line, a test can always be repeated to dispel doubts regarding an infection–before starting a potentially unrequired antibiotic treatment triggered by an excessive sensitivity and false-positive diagnostic outcome.

In terms of the detection of VlsE-specific AB, LIA C demonstrated the highest sensitivity overall; it rated the greatest number of samples as “VlsE-positive” (signal ≥ COC) followed by LIA B and LIA A. It stands out that in all three LIAs the Non-Vac-group continuously displayed the lowest number of samples rated as “VlsE-positive” in comparison to the vaccinated groups, while group Vac-Plus displayed the greatest number of samples with a VlsE signal intensity result above COC–at a considerably high extent in LIA C (up to 61% at d210). The inter-rater agreement regarding the detection of VlsE was poor to slight depending on time-point of sample collection and group, representing an unsatisfying agreement of tests on the one hand, and displaying a significant divergence in sensitivity and specificity of the three tests on the other hand. Considering that only equine sera have been used that formerly had been tested and rated as NEG or EQUIVOC with an in-house KELA and LIA, suggests that VlsE AG signal results of LIA A more likely picture reality than those of LIA B and LIA C. Nevertheless, the elevated detection rates in terms of VlsE-specific AB and, in this context, the high divergences in sensitivity and specificity of the three LIAs used, on the one hand portray the predicament considering the lack of replicability and reliability of diagnostic results across non-standardized serodiagnostic tests, and on the other hand, pose the question of its source.

Since none of the three LIAs displayed a significant increase in the number of VlsE-positive samples in any of the three study groups over the progressing sampling time-points in the observational period, it is unlikely that excessive VlsE-AB detection rates are linked to the application of lysate vaccines against *Bb*sl. Further, the possibility that the application of different LIA test kit LOTs per manufacturer may have had an impact on signal intensity results can be excluded as well, since only one LOT number per manufacturer has been applied for all samples tested. Our explanations for the disagreement of results pertaining to the detection of VlsE-specific AB and the comparatively high detection rate in group Vac-Plus are based on multiple reasons. Firstly, it seems that the Vac-Plus study group unknowingly compromised a higher number of naturally infected horses than study groups Non-Vac and Vac-Basic. Secondly, a rather small sample size per study group has been used in this project, hence, divergences in results are more striking; therefore, a greater sample size is preferable for several reasons. Thirdly, the cutoff control in LIA C seems to be poorly adjusted and too sensitive regarding the detection of VlsE-AB resulting in an unlikely high number of VlsE-positive samples. Thus, we highly recommend a revision and adjustment of LIA C in this aspect.

However, for dogs and humans it has been described that in patients with confirmed bacterial or viral infections (i. a. *Leptospira interrogans*, Epstein–Barr virus, Cytomegalovirus) certain borreliae test antigens commonly used in serodiagnostic assays for diagnosis of LB are the cause for cross-reactions to antibodies induced by other infectious agents not belonging to the genus *Borrelia* [34–38]. Hence, *Bb*sl antigens such as FlaB, but also OspC and BmpA are recognized nonspecifically by antibodies directed against other pathogens [36, 39]. Cross-reacting antibodies involve a high potential to generate false-positive test results and lead to a limited diagnostic value of a test. In consequence, a greater number of AG signal lines on a strip, particularly error-prone AGs such as p41 (FlaB), may have a negative impact on the effectiveness of a serotest. In view of the p41, only LIA C does contain it within its nine AG signal lines, while strips of LIA A, compromising seven AG signal lines, and LIA B, containing ten AG signal lines, excluded the p41 AG.

In view of overall test results of non-vaccinated and vaccinated horses, again, we recognize previously mentioned deficits of tests regarding sensitivity and specificity of single AG signals, as well as divergent interpretation of single AG lines. Those LIA-specific deficiencies resulted in a high number of false-positive overall test results, especially in vaccinated groups, and thus, in a low inter-rater agreement. Overall, by comparison of the three LIAs, LIA C demonstrated the upmost accuracy in terms of the overall diagnostic outcome.

The application of the ROEP achieved an improvement in the diagnostic accuracy, to an especially high extent in LIA A and LIA B. Next to the refined interpretation of infection- and vaccination-specific immunoreactions in equine sera, this standardized reference protocol covered borderline cases and cases that have not been specified before in LIA protocols supplied by manufacturers.

As emphasized earlier, the application of the ROEP also had influence on overall test results in the Non-Vac-group, yet only in LIA B. Application of the ROEP mainly resulted in a greater number of “EQUIVOC” samples. It suggests that these samples possibly were rated false-positive or false-negative in the first place. Hereby, an allocation to the “EQUIVOC” category is beneficial, since sample testing may be repeated and thus avoids inadequate treatment of the horse.

Since group Non-Vac functioned as non-vaccinated negative control group and was not influenced by possible effects of a vaccination, a high inter-rater agreement was expected. Yet, despite that group Non-Vac displayed the highest observed inter-rater agreement (d0: 71%, d135: 70%, d210: 63%) for overall test results at three time-points, its IRR was only slight with a low kappa ranging between 0.00 and 0.10 depending on time-point of sample collection. With application of the ROEP, the kappa significantly improved to fair at d0 and d135 (*κ* = 0.22), while the observed inter-rater agreement only slightly improved. Here it needs to be emphasized that the Fleiss’ kappa, computing the inter-rater reliability of several raters rating the same thing by calculating the ratio between observed agreement (P_o_) and agreement expected by chance (P_e_), sometimes is difficult to interpret. At times, a paradox situation may be created, where a high level of observed raters’ agreement might be represented by a low kappa value. This might be the case in group Non-Vac when displaying a high discrepancy between a high P_o_ and a low IRR. Taking this paradox into account, overall, group Non-Vac displays the highest observed agreement of the three experimental groups at all three time-points. Nevertheless, the agreement of results between raters is in need for improvement as well.

### 4.2. Handling, operability and evaluation procedure of LIAs

Handling and implementation of the three tests in the laboratory were similar with minor variations. While incubation trays of LIA A and LIA B have visibly numbered slots, LIA C’s incubation trays lack this beneficial feature. Additionally, in LIA B it is advantageous that the washing buffer develops little bubbles when serum samples are added into slots of a well; it facilitates keeping track visually during the consecutive addition of samples to slots, particularly, since LIA B´s strip numbers in a test-kit are often non-consecutive. In terms of preparing reagents for use, LIA A and LIA B require preparation of the conjugate by mixing conjugate concentrate with washing buffer; LIA C offers ready-to-use conjugate. Here, the instruction manual of LIA B seems to not have included any spare volume for the conjugate when calculating the necessary reaction volume per strip. In LIA B, the shape and the small size of the reagents’ bottles and the bottles’ mouth are disadvantageous; it is complicated and time-consuming to remove the full liquid volume of a bottle, a time-critical attribute when handling a high number of samples. Further, LIA A and LIA B feature shorter incubation times for serum and conjugate than LIA C (30 min versus 45 min). Further, the substrate incubation time is two minutes longer for LIA A compared to LIA B and LIA C (12 min versus 10 min).

LIA C offers a multi-species conjugate for both, canine and equine serum samples–a positive feature when the test is used in diagnostic laboratory receiving a variety of field samples; in contrast, test kits from LIA A and LIA B contain conjugate for only one species. In LIA A it is necessary to order the canine test kit (incl. test strips, buffer concentrate, canine COC, canine conjugate concentrate, substrate) in addition to the supplementary equine set (incl. equine COC and equine conjugate concentrate) in order to be able to test equine serum samples.

LIA strips need to be attached to an evaluation sheet. LIA B solves this issue elegantly, because the sheet surface becomes sticky when the wet LIA-strips are placed onto it. In LIA A and LIA C strips are attached to the evaluation sheet using a glue roller. In LIA A strips are evaluated visually by the examiner by comparing single AG signals on the test strip with the COC line on a separate sheet, and results are recorded manually. Thus, it is prone to human error; depending on the experience of the evaluator and light conditions the assessment might vary, and evaluation results are not equally precise and comparable as when performed with an automatic scanning and evaluation system as used in LIA B and LIA C. Here, though, evaluation sheets must be prepared in advance requiring specific software and a printer. In LIA C an individual barcode must be created for every single evaluation sheet containing up to 20 samples and test strips; the scanning and evaluation process is not feasible without functioning barcode on the sample sheet. In the case of LIA B it is advantageous that multiple evaluation sheets with up to 32 samples and test strips per sheet can be scanned, attached to each other, and evaluated by the software. After scanning, the color reaction for each AG line is displayed as a number from 0 to 255 in LIA B, and 0 to 9 in LIA C, and the sample is assessed overall by the software and allocated to an allocation category. Results can be stored in the program folder or exported as image or PDF file to an external folder. Additionally, LIA C’s results can be exported as a Microsoft Excel file, which facilitates statistical analysis.

In the authors’ opinion, the evaluation via a computerized scanning and evaluation system seems to be most suitable for the scientific field due to precise evaluation of samples and thus comparability. Also, result sheets can digitally and directly be sent to commissioning veterinarians as medical report. On the other hand, if technique fails, the personnel in the diagnostic laboratory must rely on visual evaluation, which can be challenging with strips of LIA B and LIA C since the COC line is located on the same strip as the sample AG signals.

In summary, all three LIAs showed positive and negative features in their handling and operability. In our view, LIA A and C are equally user-friendly in the laboratory use, while LIA B is preferable regarding the scanning and evaluation process.

## 5. Conclusions

Diagnosis of equine LB is a challenging venture. Serodiagnosis of LB in horses should only be applied to confirm infection with *Bb*sl in order to support clinical diagnosis. To avoid misdiagnosis of LB and mistreatment of the equine patient, it is of major importance to have practical, well applicable, reliable, highly specific serodiagnostic tests available. It should feature test strips with suitable AG signals with optimal sensitivity, high specificity, and the ability to accurately detect *Bb*sl-specific AB in serum samples of horses. Moreover, it should be able to distinguish between AB generated by a past or active natural infection as well as an immune response due to immunization. To address these key challenges and enhance diagnostic accuracy, validation and standardization of tests used for serodiagnosis of equine LB are required. In any case, practicing veterinarians and horse owners must be able to rely on the diagnostic outcome of a serodiagnostic test in order to initiate an appropriate treatment of an infected horse with a clinically apparent LB, only.

By assessing three commercial LIAs for LB diagnostics in equine serum we were able to demonstrate that the available LIAs are suitable for the purpose mentioned, yet, displayed deficiencies in sensitivity, specificity and interpretation of results leading to a limited diagnostic accuracy. Consequently, adjustments are required on several levels.

Firstly, sensitivity and specificity of single AG signals need refinement, in particular those of vaccination-specific OspA and infection-specific VlsE, and potentially other AG signals present on strips as well.

This is of major importance, because in regard to OspA a false-negative AG-AB immunocomplex signal result for OspA for vaccinated and non-infected horses may result in a false-positive diagnostic outcome. That is, possibly a certain amount of other, vaccination-induced AG-AB signals (except VlsE / C6) might appear positive, and such samples are then falsely classified as infected with LB, if the OspA signal is negative. In this context, we were able to demonstrate again, that OspA is vaccination-specific; it is not detectable in non-vaccinated horses and potentially not in some basic-vaccinated horses due to a low AB titer, yet, it is reliably detectable at a high extent at d210 in vaccinated horses undergoing a strict vaccination schedule with repeated vaccinations. In the case of VlsE AG, which are expressed by viable borreliae, only, misdiagnosis is just as dangerous for the patient. From this perspective, a readjustment and revision of LIA C is recommended due to the comparatively high detection rate of VlsE-specific AB across samples.

Secondly, it is essential to correctly interpret the OspA signal as vaccination-specific, as well as an additional variety of other AG-AB signals potentially induced by the application of lysate vaccines against LB in horses. That is, additional positive AG-AB signals (next to OspA) are possibly not indicative for an LB infection, unless VlsE / C6 signal is positive. Hereby, a revision of the technical manual of LIA A and LIA B is urgently expected and required.

With the established ROEP we provide a standard sample protocol as guideline for the evaluation and classification of equine serum samples in LIA in order to improve the diagnostic accuracy of LIAs, avoid over-diagnosis of equine LB and mistreatment of the equine patient.

Another desirable adjustment for all modern LIAs in the scientific field is the application of a computerized and automatic scanning and evaluation software as already supplied by LIA B and LIA C, but not LIA A. It speeds up the process, it is user-friendly and, most importantly, it is less prone to human error, allowing a precise evaluation of samples and thus comparability.

## Supporting information

S1 Fig Flowchart on the conduction of three line immunoassays (*LIA A, **LIA B, ***LIA C) used for semi-quantitative detection of IgG antibody response against antigens that are specific for Borrelia species of the *Bb*sl. complex. WB, washing and incubation buffer; *Aq*. *dest*., *Aqua destillata*.
(TIF)

S2 Fig LIA strips representative for vaccinated horses of group Vac-Plus displaying semi-quantitative antibody reactions against *Bb*sl-specific antigens during the observational period. Tested antigens are displayed on the left. The first LIA strip shows the COC which functions as coloration reference for the signal intensity of AG-AB immunocomplex reactions; during the evaluation process it is compared to the degree of color reaction intensity of signals on the test strips produced by serum samples tested. The following LIA strips on the right were incubated with equine sera from vaccinated horses in chronical order from d0 to d135 to d210. The color reaction intensity of the vaccination-specific OspA signal on the LIA strips is increasing over the observational period with a high at d210–30 days after the third vaccination of horses in group Vac-Plus. Vac-Plus, horses vaccinated on d0, d14 and d180; d, day; COC, cutoff control; VlsE, variable major protein-like sequence expressed; Osp, outer surface protein; DbpA, decorine binding protein A; BmpA, *Borrelia* membrane protein A; kDa, kilodalton; AG, antigen; AB, antibody.
(TIF)

S1 Table Serostatus of horses used in this study. (DOCX)

S2 Table Line immunoassays used in this study. (DOCX)

S3 Table Inter-rater agreement in OspA and VlsE AG-AB immunocomplex signal intensity results at three time-points of blood collection–calculation of observed inter-rater agreement (P_o_) and statistic inter-rater reliability (IRR), represented by Fleiss’ kappa coefficient (κ). (DOCX)

S4 Table Inter-rater agreement in overall test results at three time-points of blood collection–calculation of observed inter-rater agreement (P_o_) and statistic inter-rater reliability (IRR), represented by Fleiss’ kappa coefficient (κ). (DOCX)

S5 Table LIA B—group Non-Vac–divergent allocation results for equine serum samples in the non-vaccinated group due to application of the alternative recommended overall evaluation protocol (ROEP). (DOCX)

S6 Table Abbreviations. (DOCX)
