# Supplementary figures and images for: A comparative study evaluating three line immunoassays available for serodiagnosis of equine Lyme borreliosis: Detection of Borrelia burgdorferi sensu lato-specific antibodies in serum samples of vaccinated and non-vaccinated horses
Source: PLoS One. 2024 Dec 23;19(12):e0316170. doi: 10.1371/journal.pone.0316170 (PMC11666002; doi:10.1371/journal.pone.0316170)

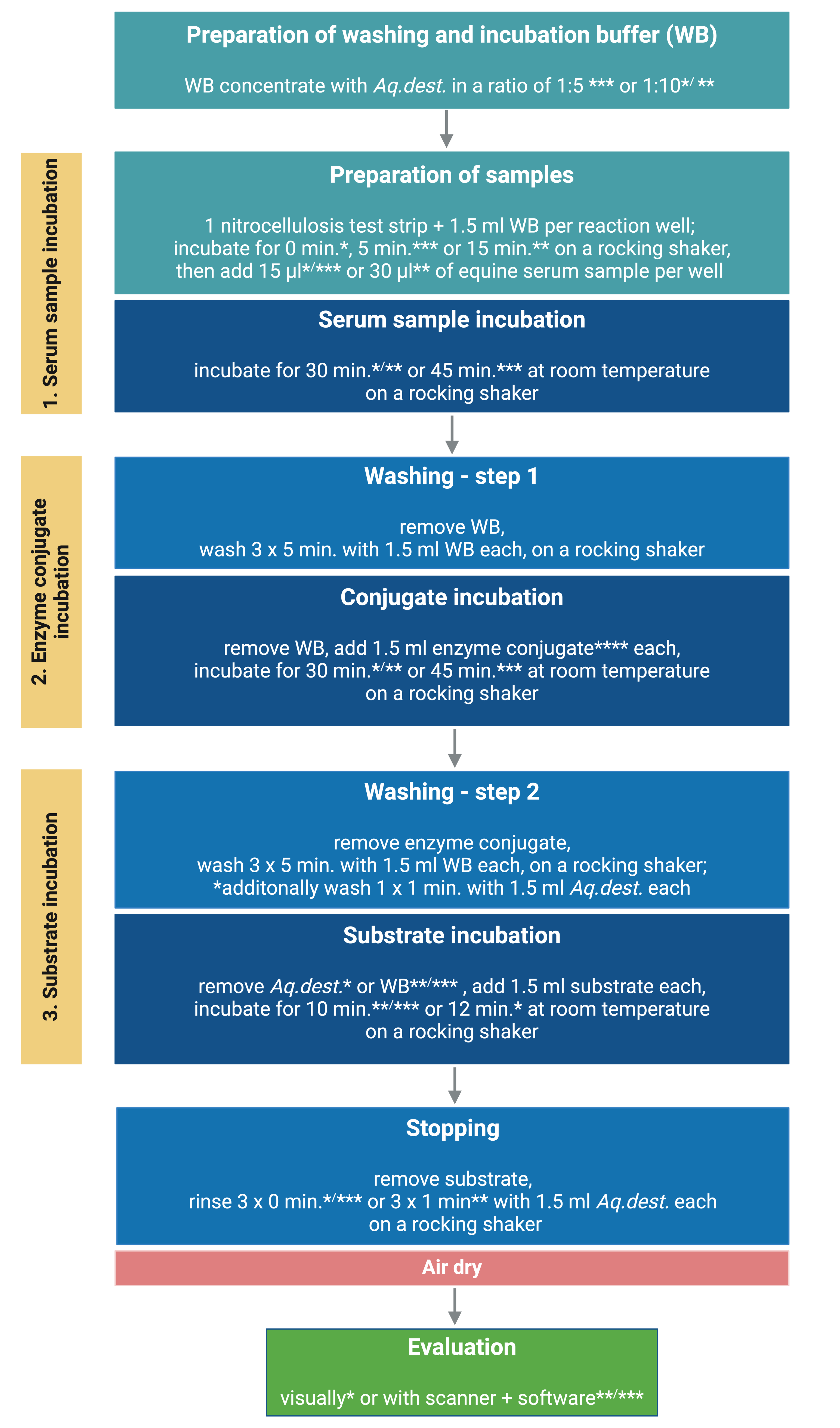

Supplement: S1 Fig — WB, washing and incubation buffer; Aq. dest., Aqua destillata. (TIF) [file pone.0316170.s001.tif]

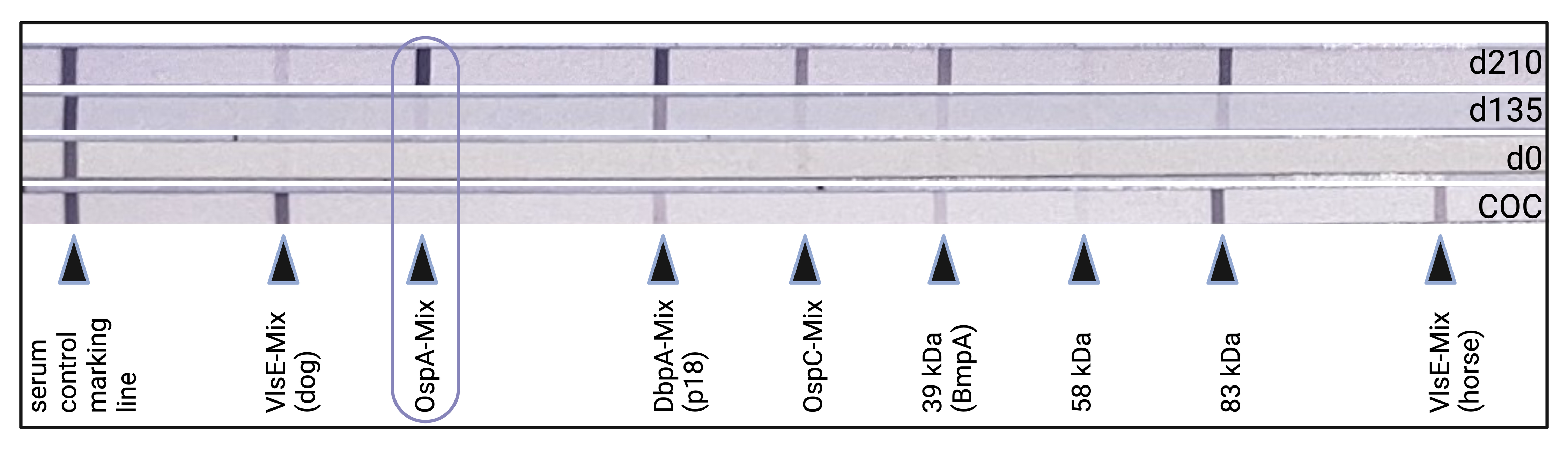

Supplement: S2 Fig — Tested antigens are displayed on the left. The first LIA strip shows the COC which functions as coloration reference for the signal intensity of AG-AB immunocomplex reactions; during the evaluation process it is compared to the degree of color reaction intensity of signals on the test strips produced by serum samples tested. The following LIA strips on the right were incubated with equine sera from vaccinated horses in chronical order from d0 to d135 to d210. The color reaction intensity of the vaccination-specific OspA signal on the LIA strips is increasing over the observational period with a high at d210–30 days after the third vaccination of horses in group Vac-Plus. Vac-Plus, horses vaccinated on d0, d14 and d180; d, day; COC, cutoff control; VlsE, variable major protein-like sequence expressed; Osp, outer surface protein; DbpA, decorine binding protein A; BmpA, Borrelia membrane protein A; kDa, kilodalton; AG, antigen; AB, antibody. (TIF) [file pone.0316170.s002.tif]
