# Supplementary material for: A comparative study evaluating three line immunoassays available for serodiagnosis of equine Lyme borreliosis: Detection of Borrelia burgdorferi sensu lato-specific antibodies in serum samples of vaccinated and non-vaccinated horses
Source: PLoS One. 2024 Dec 23;19(12):e0316170. doi: 10.1371/journal.pone.0316170 (PMC11666002; doi:10.1371/journal.pone.0316170)
Supplement: S1 Table — (DOCX) [file pone.0316170.s003.docx]

**S1 Table.** **Serostatus of horses used in this study**.

| **Serostatus of equine sera / group** | **Non-Vac** | **Vac-Basic** | **Vac-Plus** |
| --- | --- | --- | --- |
| **Number of participating horses (*n* = 131)** | 42 | 45* | 44* |

*Horses experimentally vaccinated with the vaccine EquiLyme® (Boehringer Ingelheim Vetmedica GmbH, Ingelheim am Rhein, Germany (13)). Non-Vac, non-vaccinated horses; Vac-Basic, horses vaccinated on day 0 (d0) and day 14 (d14); Vac-Plus, horses vaccinated on day 0 (d0), day 14 (d14) and day 180 (d180).
