# Supplementary material for: A comparative study evaluating three line immunoassays available for serodiagnosis of equine Lyme borreliosis: Detection of Borrelia burgdorferi sensu lato-specific antibodies in serum samples of vaccinated and non-vaccinated horses
Source: PLoS One. 2024 Dec 23;19(12):e0316170. doi: 10.1371/journal.pone.0316170 (PMC11666002; doi:10.1371/journal.pone.0316170)
Supplement: S2 Table — (DOCX) [file pone.0316170.s004.docx]

**S2 Table.** **Line immunoassays used in this study.**

| **LIA** | **LIA A** | **LIA B** | **LIA C** |
| --- | --- | --- | --- |
| **Manufacturer/ test kit** | VIROTECH  Borrelia Vet. + OspA IgG LINE Immunoblot, Germany | EUROIMMUN Anti-Borrelia-EUROLINE horse (IgG), Germany | MEGACORMegaLINE® BORRELIA IgG ad.us.vet. LINE immunoassay, Austria |
| **Kit LOT number** | 202-0805 (horse-kit),  202-1104 (dog-kit) | D220815AC - 2024/01 | ML-B-012 - 2024/05 |

LIA, line immunoassay; IgG, immunoglobulin G.
