# Supplementary material for: A comparative study evaluating three line immunoassays available for serodiagnosis of equine Lyme borreliosis: Detection of Borrelia burgdorferi sensu lato-specific antibodies in serum samples of vaccinated and non-vaccinated horses
Source: PLoS One. 2024 Dec 23;19(12):e0316170. doi: 10.1371/journal.pone.0316170 (PMC11666002; doi:10.1371/journal.pone.0316170)
Supplement: S3 Table — (DOCX) [file pone.0316170.s005.docx]

**S3 Table. Inter-rater agreement in OspA and VlsE AG-AB immunocomplex signal intensity results at three time-points of blood collection – calculation of observed inter-rater agreement (P_o_) and statistic inter-rater reliability (IRR), represented by Fleiss’ kappa coefficient (κ).**

| **Time-point blood sample**  **collection** | **Experimental group** | **P_o_ for OspA AG line** | **κ for OspA AG line** | | **P_o_ for VlsE AG line**  **–** | **κ for VlsE AG line** | |
| --- | --- | --- | --- | --- | --- | --- | --- |
|  | **Vac-Basic** | 69% | 0.10 | slight | 36% | -0.07 | poor |
| **d0** | **Vac-Plus** | 68% | 0.05 | slight | 39% | 0.07 | slight |
|  | **Non-Vac** | 63% | 0.09 | slight | 38% | -0.16 | poor |
|  | **Vac-Basic** | 50% | 0.06 | slight | 33% | -0.05 | poor |
| **d135** | **Vac-Plus** | 53% | 0.13 | slight | 40% | 0.09 | slight |
|  | **Non-Vac** | 56% | -0.04 | poor | 31% | -0.15 | poor |
|  | **Vac-Basic** | 61% | 0.30 | fair | 31% | -0.07 | poor |
| **d210** | **Vac-Plus** | 95% | -0.02 | poor | 32% | -0.01 | poor |
|  | **Non-Vac** | 70% | 0.10 | slight | 34% | -0.09 | poor |

The P_o_ and IRR, represented by *κ*, are calculated per time-point of blood collection with subdivision into experimental groups (Vac-Basic, Vac-Plus, Non-Vac). The IRR was categorized into “poor” (*κ* = < 0.0), “slight” (*κ* = 0.0 to 0.2), “fair” (*κ* = 0.21 to 0.40), “moderate” (*κ* = 0.41 to 0.60), “substantial” (*κ* = 0.61 to 0.80), and “almost perfect” (*κ* = 0.81 to 1.00). A high value for *κ* represents a high agreement of the three tests or raters regarding a sample`s overall result, or its coloration of single AG signals respectively.

OspA, outer surface protein A; VlsE, variable major protein-like sequence expressed; AG, antigen; AB, antibody; P_o_, observed inter-rater agreement; IRR, statistic inter-rater reliability; κ, Fleiss’ kappa coefficient; d, day; Non-Vac, non-vaccinated horses; Vac-Basic, horses vaccinated on d0 and d14; Vac-Plus, horses vaccinated on d0, d14 and d180.
