# Supplementary material for: A comparative study evaluating three line immunoassays available for serodiagnosis of equine Lyme borreliosis: Detection of Borrelia burgdorferi sensu lato-specific antibodies in serum samples of vaccinated and non-vaccinated horses
Source: PLoS One. 2024 Dec 23;19(12):e0316170. doi: 10.1371/journal.pone.0316170 (PMC11666002; doi:10.1371/journal.pone.0316170)
Supplement: S4 Table — (DOCX) [file pone.0316170.s006.docx]

**S4 Table.** **Inter-rater agreement in overall test results at three time-points of blood collection – calculation of observed inter-rater agreement (P_o_) and statistic inter-rater reliability (IRR), represented by Fleiss’ kappa coefficient (κ).**

| **Time-point blood sample**  **collection** | **Experimental group** | **P_o_ for overall results – after evaluation according to manufacturer’s instructions** | | **κ for overall results – after evaluation according to manufacturer’s instructions** | | **P_o_ for overall results – after evaluation according to ROEP** | | **κ for overall results – after evaluation according to ROEP** | |
| --- | --- | --- | --- | --- | --- | --- | --- | --- | --- |
| **d0** | **all groups** | 66% | 0.28 | | fair | 68% | 0.35 | | fair |
|  | **Vac-Basic** | 67% | 0.28 | | fair | 68% | 0.33 | | fair |
|  | **Vac-Plus** | 60% | 0.32 | | fair | 63% | 0.38 | | fair |
|  | **Non-Vac** | 71% | 0.06 | | slight | 72% | 0.22 | | fair |
| **d135** | **all groups** | 55% | 0.18 | | slight | 54% | 0.24 | | fair |
|  | **Vac-Basic** | 50% | 0.15 | | slight | 48% | 0.19 | | slight |
|  | **Vac-Plus** | 47% | 0.18 | | slight | 45% | 0.20 | | fair |
|  | **Non-Vac** | 70% | 0.10 | | slight | 71% | 0.22 | | fair |
| **d210** | **all groups** | 41% | 0.18 | | slight | 59% | 0.43 | | moderate |
|  | **Vac-Basic** | 44% | 0.11 | | slight | 44% | 0.18 | | slight |
|  | **Vac-Plus** | 17% | -0.23 | | poor | 67% | 0.33 | | fair |
|  | **Non-Vac** | 63% | 0.00 | | slight | 66% | 0.17 | | slight |

First, evaluation was performed according to manufacturers’ instructions (results on the left) and, second, according to ROEP (results on the right). The P_o_ and IRR, represented by *κ*, are calculated per time-point of blood collection – once without (all groups), and second, with additional subdivision into experimental groups (Vac-Basic, Vac-Plus, Non-Vac). The IRR was categorized into “poor” (*κ* = < 0.0), “slight” (*κ* = 0.0 to 0.2), “fair” (*κ* = 0.21 to 0.40), “moderate” (*κ* = 0.41 to 0.60), “substantial” (*κ* = 0.61 to 0.80), and “almost perfect” (*κ* = 0.81 to 1.00). A high value for *κ* represents a high agreement of the three tests or raters regarding a sample`s overall result, or its coloration of single AG signals respectively.

ROEP, recommended overall evaluation protocol; P_o_, observed inter-rater agreement; IRR, statistic inter-rater reliability; κ, Fleiss’ kappa coefficient; d, day; Non-Vac, non-vaccinated horses; Vac-Basic, horses vaccinated on d0 and d14; Vac-Plus, horses vaccinated on d0, d14 and d180. Non-Vac, non-vaccinated horses; Vac-Basic, horses vaccinated on d0 and d14; Vac-Plus, horses vaccinated on d0, d14 and d180.
