# Supplementary material for: A comparative study evaluating three line immunoassays available for serodiagnosis of equine Lyme borreliosis: Detection of Borrelia burgdorferi sensu lato-specific antibodies in serum samples of vaccinated and non-vaccinated horses
Source: PLoS One. 2024 Dec 23;19(12):e0316170. doi: 10.1371/journal.pone.0316170 (PMC11666002; doi:10.1371/journal.pone.0316170)
Supplement: S5 Table — (DOCX) [file pone.0316170.s007.docx]

**S5 Table.** **LIA B - group Non-Vac – divergent allocation results for equine serum samples in the non-vaccinated group due to application of the alternative recommended overall evaluation protocol (ROEP).**

| **Allocation result –**  **evaluation according to manufacturer** | **Allocation**  **result –**  **evaluation**  **according to ROEP** | **Number of samples with divergent**  **result at d0**  **[*n*]** | **Number of samples with divergent**  **result at d135**  **[*n*]** | **Number of samples with divergent**  **result at d210**  **[*n*]** | **Case Example**  **(group Non-Vac)** |
| --- | --- | --- | --- | --- | --- |
| POS | EQUIVOC | 2 | 1 | 1 | 3 AG lines (not VlsE / OspA) ≥ COC |
| NEG | EQUIVOC | 5 | 4 | 5 | VlsE AG line ≥ COC  **or**  VlsE and p100 AG lines ≥ COC  **or**  VlsE AG line ≥ COC and 1 AG line = COC  **or**  ≤ 2 AG lines (incl. VlsE) = COC |
| EQUIVOC | POS | 0 | 1 | 1 | 1 AG line ≥ COC and 3 AG lines = COC  **or**  2 AG lines ≥ COC and 2 AG lines (incl. VlsE) = COC |

Evaluation and allocation of LIA strips was performed once according to manufacturer’s instructions, and second, according to ROEP. The table shows the number of divergent allocation results for LIA B in group Non-Vac at a certain time-point. Only in LIA B there is a discrepancy in the allocation results in group Non-Vac; for LIA A and LIA C overall results for this group remained unchanged, independent of the evaluation approach and time-point, and therefore, are not included in this table.

Non-Vac, non-vaccinated horses; ROEP, recommended overall evaluation protocol; d, day of blood sample collection during the vaccination schedule; NEG, negative overall test result; EQUIVOC, equivocal overall test result; POS, positive overall test result (infection); AG, antigen; OspA, outer surface protein A; VlsE, variable major protein-like sequence expressed; COC, cut off control.
