# Supplementary material for: A comparative study evaluating three line immunoassays available for serodiagnosis of equine Lyme borreliosis: Detection of Borrelia burgdorferi sensu lato-specific antibodies in serum samples of vaccinated and non-vaccinated horses
Source: PLoS One. 2024 Dec 23;19(12):e0316170. doi: 10.1371/journal.pone.0316170 (PMC11666002; doi:10.1371/journal.pone.0316170)
Supplement: S6 Table — (DOCX) [file pone.0316170.s008.docx]

**S6 Table.** **Abbreviations**

| °C | degrees Celsius |
| --- | --- |
| AB | antibody |
| AG | antigen |
| *Aq. dest.* | *Aqua destillata* |
| *Ba* | *Borrelia afzelii* |
| *Bb* | *Borrelia burgdorferi* |
| *Bb*sl | *Borrelia burgdorferi* sensu lato |
| BmpA | *Borrelia* membrane protein A |
| C6 | peptide referring to 6th invariant region of VlsE |
| COC | cut off control line |
| d | day |
| DbpA | Decorine binding protein A |
| ELISA | enzyme-linked immunosorbent assay |
| equivoc | equivocal |
| ErpA | A-type iron sulfur protein essential for respiratory metabolism in *Escherichia coli* |
| i. a. | inter alia; among other things |
| i. e. | id est; in other words |
| IFA | Indirect fluorescent antibody test |
| IgG | Immunoglobulin gamma |
| *k* | Kappa |
| kDa | Kilodalton |
| KELA | kinetic ELISA |
| L | lipid |
| LB | Lyme borreliosis |
| LIA | line immunoassay |
| μl | microlitre(s) |
| min | minute(s) |
| ml | millilitre(s) |
| *n* | natural number |
| neg | negative |
| np | not present |
| Osp | outer surface protein |
| p | protein |
| pos | positive |
| ROEP | recommended overall evaluation protocol |
| TBD | Tick-borne disease |
| Vac | vaccinated |
| VlsE | Variable major protein like sequence expressed |
